# 

**Published:** 1960-07

**Authors:** 


### Contents

MOUNTAINS . . . . . *Dr. F. W. Terrell Hughes* 43

MANAGEMENT OF SEPSIS . . . *Herbert K. Bourns* 50

X-RAYS IN EVERYDAY LIFE . . . *J. H. Middlemiss* 55

NEPHROTIC SYNDROME WITH STEATORRHOEA . . . *Clinical Pathological Conference* 64

NOTES AND NEWS . . . . . . . . 74

EXAMINATION RESULTS . . . . . . 74

PRIZES . . . . . . . . . 75

APPOINTMENTS . . . . . . . . . 75

